# Targeted Adversarial Camouflage Texture for Fooling Object Detectors via Native Supervision Redirection

**DOI:** 10.3390/e28070718

**Published:** 2026-06-24

**Authors:** Xingyu Di, Wei Cai, Xin Wang, Zhongjie Yin, Shuhui Li, Haoran Jia

**Affiliations:** College of Missile Engineering, Rocket Force University of Engineering, Xi’an 710025, China; dixy1998@outlook.com (X.D.); wangxin9550@outlook.com (X.W.); yin503777019@163.com (Z.Y.); lsh_1217@foxmail.com (S.L.); rq159357@outlook.com (H.J.)

**Keywords:** physical adversarial attack, adversarial camouflage, targeted attack, object detection, empirical evaluation

## Abstract

Adversarial camouflage has attracted growing research attention owing to its ability to execute multi-view, persistent attacks in real physical environments, outperforming conventional single-view adversarial patches. However, most existing methods are confined to non-targeted attacks, which induce arbitrary incorrect detection results without specifying target categories. This ambiguity weakens attack destructiveness and stealthiness, posing limitations for security evaluation of real-world vision systems. To address this gap, we present TACT, an approach built upon the full-coverage physical camouflage pipeline. By replacing the original category supervision with a predefined target class, TACT redirects the optimization gradient to guide 3D texture toward the target category features. Such a scheme only employs the inherent feature alignment mechanism of off-the-shelf object detectors, without redesigning network modules, defining novel loss functions, or modifying the rendering pipeline. Extensive experiments across digital and physical domains validate its effectiveness: on seven mainstream general-purpose object detectors, TACT-person achieves an average targeted attack success rate of 51.91%, and delivers cross-architecture and cross-version transferability. In physical tests, TACT-bird reduces mAP50-95 by 59.87% on YOLOv8, yet a TCER–TASR gap suggests that the physical pipeline acts as a low-pass filter: coarse-grained target classes transfer robustly while fine-grained ones suffer feature collapse. These results confirm the viability of native supervision redirection and reveal an empirical pattern: coarse-grained target classes transfer more robustly through the physical pipeline than fine-grained ones, suggesting that target class feature granularity consistently influences physical-domain attack effectiveness.

## 1. Introduction

**Figure 1:**
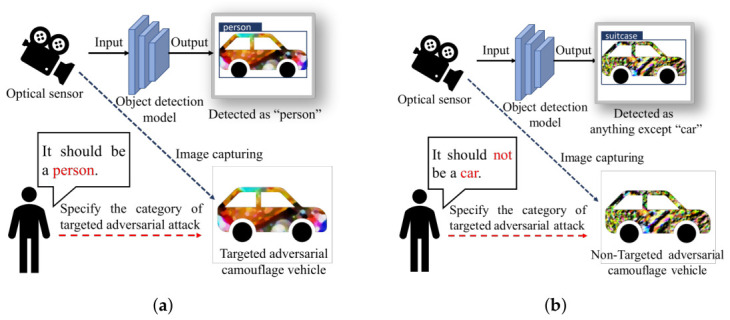
The difference between targeted attacks and non-targeted attacks in adversarial attacks. (**a**) Specified as the category “person”, adversarial camouflage will cause the detector to misclassify the target as “person”. (**b**) Not specified category, the adversarial camouflage will cause the detector to misclassify it as any category other than “car”, or fail to detect any object.

The increasing reliance on deep learning-based object detectors in safety-critical cyber-physical systems [1], such as autonomous vehicles and surveillance drones, has made their robustness a paramount security concern [2]. Among various threats, physical adversarial attacks pose a particularly severe risk [3], as they can induce persistent, real-world misperception by manipulating an object’s appearance [4]. While most research has focused on non-targeted attacks, targeted attacks maliciously misclassify an object into a specific, attacker-chosen category (e.g., “car” → “person”). Both attack types pose substantial safety risks to cyber-physical systems. In particular, such targeted attacks may directly trigger predefined incorrect system decisions, thereby fundamentally compromising operational safety and security [5,6]. The distinction between these two attack modes is illustrated in Figure 1.

To realize robust targeted physical adversarial attacks, a practical physical targeted attack needs to meet three core conditions: (1) physical realizability: The adversarial pattern must be manufacturable and applicable to complex, full-coverage 3D objects like vehicles [7]; (2) attack specificity: It should reliably cause misclassification into a predefined target category, rather than inducing arbitrary errors [8]; and (3) model-agnostic transferability: It should remain effective against black-box deployed models whose internal parameters and architectures are unknown [9]. Early patch-based methods [10] lack full coverage and multi-view robustness. While recent full-coverage camouflages [11,12] have made significant progress, they are predominantly non-targeted. Although initial explorations into targeted physical attacks exist [8,13], they frequently rely on complex, handcrafted loss functions or auxiliary networks, which introduce significant optimization overhead and can obscure the fundamental attack mechanism. This landscape reveals a critical gap: the absence of a simple and efficient method to generate targeted adversarial camouflage that directly probes a model’s vulnerability to feature-space manipulation.

We posit that the key to bridging this gap lies not in designing more complex optimization objectives, but in repurposing the model’s inherent training objective. Modern object detectors are optimized with loss functions that naturally establish feature-label alignment. This work introduces a strategy of native supervision redirection for targeted adversarial texture generation. This operation effectively redirects the gradient flow derived from the detector’s original, unmodified loss function. It drives the optimizer to synthesize surface textures that shift the object’s extracted features toward the latent distribution of the target class, thereby achieving targeted misclassification by “hijacking” the model’s native learning signal. This finding reveals a fundamental vulnerability: the intrinsic training mechanics of object detectors can be readily repurposed to craft adversarial camouflage that misleads the model itself.

Based on this insight, we present Targeted Adversarial Camouflage Texture (TACT), an approach for generating full-coverage, multi-view robust, and target-specific adversarial textures for 3D vehicles. TACT requires no modification to the victim detector’s architecture or loss function, and introduces no auxiliary networks. Extensive experiments demonstrate that textures generated by TACT achieve high targeted attack success rates across a wide spectrum of black-box detectors (including YOLO series, Faster R-CNN, and SSD) in digital simulation. Crucially, we validate their physical realizability by fabricating and testing these camouflages on scale-model vehicles, showing a significant degradation of real-world detection performance. Beyond attack effectiveness, our physical experiments reveal an empirical pattern suggesting that the printing–capture pipeline exhibits low-pass filter-like behavior: the spatial frequency of the target class’s discriminative features consistently influences physical-domain targeted attack viability. Specifically, the main contributions of this study can be summarized as follows:

- We present a native supervision redirection strategy for targeted physical adversarial camouflage. Unlike existing physical adversarial camouflage methods, including non-targeted approaches such as FCA [11], RAUCA [14], and ACTIVE [9], which rely on custom-designed loss functions, TACT directly repurposes the detector’s native, unmodified loss function by redirecting the classification supervision label toward a predefined target category. This challenges the prevailing assumption that effective adversarial texture optimization in the physical domain requires loss function redesign, and demonstrates that the detector’s inherent feature alignment mechanism is itself sufficient to synthesize targeted adversarial textures through the full rendering pipeline without introducing auxiliary networks or modifying the rendering pipeline. Unlike digital-domain targeted approaches based on imperceptible pixel-level perturbations, TACT optimizes full-coverage 3D vehicle textures that preserve targeted misclassification under real-world deployment conditions, enabling practical evaluation of targeted threats against real-world detection systems.
- We provide an extensive empirical validation of TACT across nine state-of-the-art camouflage baselines (with raw texture and random noise included as references), nine mainstream object detectors spanning three architectural families (YOLO series, two-stage R-CNN variants, SSD, and Transformer-based RT-DETR), and both digital and physical domains. Physical experiments across five viewing angles confirm that targeted misclassification survives the digital-to-physical transition, with TACT-person achieving 88.60% TASR at 90° on Mask R-CNN. We further formalize TASR and TCER as dedicated evaluation metrics that distinguish targeted misclassification from general detection failure in physical adversarial attack assessment.
- We reveal two properties of object detectors’ native loss functions through ablation and CAM visualization experiments, both of which are enabled by—and provide mechanistic support for—the native supervision redirection mechanism. First, the supervision mechanism is direction-agnostic: the same unmodified loss function can either reinforce original category features or forge target category features depending solely on the supervision label. While this property is consistent with the symmetric nature of cross-entropy, it has not been previously demonstrated in the physical adversarial camouflage setting, where gradient propagation traverses a neural renderer and a physical transformation function rather than operating directly on image pixels. Second, we reveal and experimentally validate an empirical pattern across target categories: target classes with fine-grained, high-frequency discriminative features (e.g., bird) exhibit lower physical-domain TASR than those with coarse-grained features (e.g., person). We provide convergent evidence through low-pass filtering simulation and detection-head weight-space projection, demonstrating that this asymmetry is caused by the differential vulnerability of high-frequency features to the printing–capture pipeline’s low-pass filtering effect.

## 2. Related Work

With the rapid advancement and widespread deployment of adversarial example attacks, computer vision-based adversarial techniques have evolved along three key dimensions: from digital to physical domains, single-view to multi-view attack scenarios, and unpredictable to controllable attack outcomes. This section systematically reviews state-of-the-art research on physical adversarial attacks for object detection and targeted attack methods.

### 2.1. Physical Adversarial Attack

**Figure 2:**
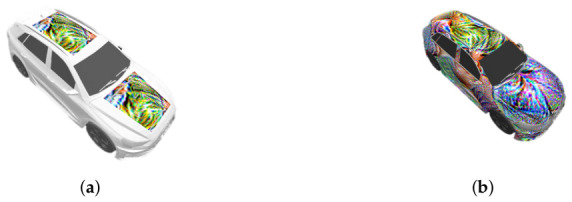
The difference between adversarial patch and adversarial camouflage. Form (**a**) corresponds to the patch-based adversarial attack, while form (**b**) refers to the camouflage-based adversarial attack.

Performing adversarial attacks on objects with non-planar and complex geometries has long been a central research challenge in the computer vision community. To address the challenges inherent in real-world deployment, researchers have extensively explored the generation of physical adversarial examples. Unlike digital attacks that apply constrained pixel-level perturbations to images [15], physical attacks induce unconstrained perturbations by modifying real-world object surface characteristics (e.g., printed patterns, structural alterations). More recently, non-contact perturbation modalities have also been introduced, including projection-based methods [16,17,18,19] and electromagnetic interference [20]. However, most such methods only work effectively on simple-shaped, uniform-surface targets (e.g., stop signs) and lack sustained multi-view robustness against detectors, especially for complex-surface objects like vehicles. Additionally, projection or electromagnetic interference-based attacks impose strict environmental requirements, limiting their deployment scenarios. Accordingly, this section focuses on persistent, directly deployable adversarial perturbation methods applicable to target objects in diverse scenarios, namely adversarial patches and adversarial camouflage. Figure 2 illustrates the differences between them.

#### 2.1.1. Patch-Based Attack

Early physical adversarial attacks relied on adversarial patches. Deployed on real-world targets, these patches alter object features, perturb sensor-captured images, and induce model mispredictions. Initially applied to image classification [21], patch-based methods were later extended to object detection and segmentation tasks [22,23,24,25,26]. However, patch-based attacks face two critical limitations: high visual saliency and strong viewpoint dependence, where minor perspective shifts or partial patch occlusion often nullify attack effects. While efforts to improve stealth (e.g., printing patches on clothing [10,27] or attaching stickers [28,29]) have been made, they sacrifice attack performance and limit practical applicability. Accordingly, to address the challenges posed by complex environmental variations and diverse imaging conditions in real-world settings, an increasing number of studies have shifted their focus to physical adversarial camouflage, and it is the attack form adopted in our work. Researchers have targeted objects with complex geometries and irregular surfaces as camouflage subjects, such as vehicles.

#### 2.1.2. Camouflage-Based Attack

Research on physical adversarial camouflage for complex 3D targets has evolved through three distinct phases, with key advancements centered on rendering differentiability and robustness. Early methods, such as CAMOU [30] and Enlarge-and-Repeat (ER) [31], laid the foundation for full-coverage camouflage but relied on non-differentiable simulators, limiting optimization efficiency. To address this critical limitation, a second wave of methods leveraged Kato et al.’s neural 3D mesh renderer, integrating differentiable rendering into network training to enable end-to-end gradient computation. Representative works include Dual Attention Suppression (DAS) [7], Full-coverage Camouflage Attack (FCA) [11], and Multi-layer Feature-aware Attack (MFA) [32], marking the “neural rendering era” of adversarial camouflage. Differentiable Transformation Attack (DTA) [12] and Adversarial Camouflage for Transferable and Intensive Vehicle Evasion (ACTIVE) [9] use differentiable transformation networks to learn scene attributes from photorealistic renderers, Robust and Accurate UV map-based Camouflage Attack (RAUCA) [14] implements UV map-based end-to-end optimization and incorporates dynamic environmental factors into rendering pipelines, resolving accuracy and robustness deficiencies of conventional and early neural rendering methods.

Despite these advancements, a critical gap remains: the vast majority of existing camouflage-based methods are confined to non-targeted attacks, which only induce arbitrary misclassifications without specifying target categories. This limits their utility for security evaluations that require controllable, malicious attack outcomes—an essential consideration for real-world applications such as autonomous driving.

### 2.2. Targeted Attack

Research on targeted attacks is relatively limited compared with that on non-targeted attacks and is mostly confined to the digital domain. Unlike non-targeted attacks that merely trigger erroneous outputs, targeted attacks exhibit clearer adversarial intent, higher implementation complexity, and stronger practical impact. Transferable Targeted Adversarial Attack (TTAA) [33] captures the distribution information of target classes from both the target and label perspectives to generate highly transferable adversarial examples. However, their work remains confined to crafting imperceptible adversarial perturbations on digital images, thus lacking physical-domain validity. Similar to approaches such as Transferable Targeted Perturbations (TTP) [34] and Minimizing the Maximum Model Discrepancy (M3D) [35], TTAA necessitates the training of auxiliary networks, which incurs substantial time costs and imposes heavy computational overhead. Zhao et al. [36] attributed the poor performance achieved by directly using non-targeted transferable methods to low attack convergence; they further found that increasing the number of iterations yields improved performance in targeted transferable attacks.

Targeted physical adversarial attacks, which enforce specific misclassifications, are essential for evaluating worst-case security vulnerabilities but remain challenging. Existing methods such as Transferable Targeted 3D (TT3D) [8] introduce significant complexity. While Multi-view Consistent adversarial Camouflage (MCC) [13] generates category-aware camouflage, it relies on integrating semantic guidance via the category mapping layer and latent synthetic network, which increases optimization overhead and may fail to guarantee robustness in complex real-world scenarios. Unlike these approaches, TACT does not introduce any additional loss terms, auxiliary networks, or semantic guidance modules—the detector’s original, unmodified loss function itself is sufficient to drive targeted texture synthesis. This distinction is not merely a matter of simplicity: it demonstrates that the targeted feature alignment objective is already embedded in the pre-trained detector’s learning signal, and no specialized optimization machinery is required to extract it.

In contrast, our TACT approach produces targeted adversarial camouflage textures directly through native supervision redirection. It avoids redefining loss functions, training additional auxiliary networks, or consuming extra computational resources. This design supports efficient and practical targeted attacks in physical environments, while preserving full-coverage deployment and differentiable optimization, thereby overcoming the key limitations of existing methods.

Table 1 summarizes a comparison among existing adversarial camouflage methods. As shown, TACT is the only approach that simultaneously satisfies all three properties: full coverage, differentiable rendering, and targeted attack capability.

## 3. Method

In this section, we define the threat model and problem statement. Subsequently, we provide an overview of our TACT approach and elaborate on the detailed design of its constituent components.

### 3.1. Threat Model

This work targets visible-light deep learning (DL) object detectors deployed in real-world environments. Specifically, we focus on the targeted attack against object detection, which refers to misclassifying one object as another. The attack is executed prior to the camera imaging process: rather than injecting perturbations into the digital images post-capture, adversarial patterns are applied directly to the physical surface of the target object. To achieve this attack objective, we assume that the attacker possesses prior knowledge of typical object detectors (e.g., earlier versions of YOLO), including their training parameters and model architectures. We optimize the adversarial camouflage texture via white-box gradient-based training on a surrogate detector (YOLOv3) with full knowledge of its architecture and weights. In real-world deployment, the optimized texture is used to perform black-box transfer attacks against deployed detectors with unknown parameters, relying on the cross-model transferability of adversarial patterns.

In this work, we target general-purpose object detection models. These models are typically trained on large-scale datasets such as MS COCO [37] and ImageNet [38], with weights optimized over hundreds of thousands to millions of annotated instances spanning diverse object categories, scenes, and viewpoints. Compared with single-task counterparts, models trained on large-scale datasets encode substantially richer visual representations and exhibit stronger generalization to real-world diversity. Consequently, mounting successful adversarial attacks against such models is more challenging, and any successful attack carries greater practical significance.

### 3.2. Problem Definition

Given a DNN object detection F(·) with parameter $\theta_{f}$, detection weight *w*, a clean example *x* without any perturbation, the ground-truth label *y* of example *x*, the dataset X that x belongs to and the label set Y of dataset X, x∈X, y∈Y. *x* is a 2D image, $x\in R^{H\times W\times 3}$. If *x* is input into F(·) for detection, the object detector can accurately detect the position and classification of the clean example *x*, and the formula is expressed as

(1) F(x)=y

Given the adversarial example x* with added targeted adversarial perturbation, where the specified class label is $y_{t}$ and $y_{t}\neq y$. When x* is fed into the detection model F(·), the model fails to correctly detect the position or classify the targeted adversarial example x*, which is denoted as

(2) $$F\left(x*\right)=y_{t}\;s.t.\;y_{t}\neq y$$

Let (X,Y,Θ) denote the multi-view training dataset; **Y** is the set of ground-truth labels corresponding to **X**, and Θ represents the collection of position and pose information (including both vehicles and cameras). We are also given a 3D vehicle model M a neural renderer R, and initial random noise $z_{0}$. A binary mask *m* (where $m\in R^{H\times W\times 1}$) is used to isolate the target vehicle region from the background in image *x* (with x∈X). The initial random noise $z_{0}$ is iteratively optimized to yield the final adversarial camouflage texture. Based on the position and pose information θ (where θ∈Θ) in the original dataset, the neural renderer R renders the adversarial camouflage vehicle model $M_{\mathrm{adv}}$ (which maps the adversarial camouflage texture $T_{\mathrm{adv}}$) into a 2D adversarial camouflage vehicle image $I_{\mathrm{adv}}$ via the face index file, and composites it into the original image scene. A physical transformation function Φ is employed to bridge the gap between the digital domain and the physical domain, generating the adversarial example $x_{\mathrm{adv}}$. This process is formulated as:

(3) $$x_{\mathrm{adv}}=\Phi \left(I_{\mathrm{adv}},m,x\right)=m\cdot T\left(I_{\mathrm{adv}}\right)+\left(1-m\right)\cdot x,$$

Here, *m* is a binary mask with value 1 for the target vehicle region and 0 for the background, T denotes a translation transformation applied to the rendered image to ensure proper spatial alignment with the original scene, avoiding abrupt black boundary artifacts. When the generated adversarial example $x_{\mathrm{adv}}$ is fed into the target detection model F(·) for detection, the detector’s output is obtained as $O=F(x_{\mathrm{adv}},\theta_{f})$, where *F* denotes the target detection model with parameter $\theta_{f}$. If the function F(·) misclassifies the adversarial vehicle target $M_{\mathrm{adv}}$ into the specified category $y_{t}$, the attack is successful.

### 3.3. Overview

**Figure 3:**
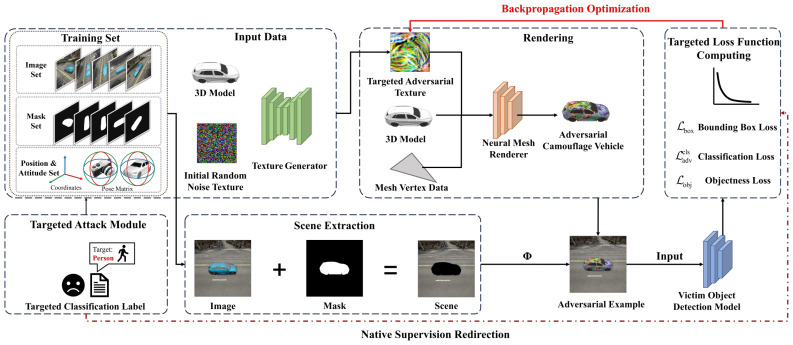
The overview of our TACT framework.

In this section, we outline the TACT framework and illustrate its core mechanism through pseudocode and a pipeline diagram. TACT is grounded in the theory of feature distribution alignment. To train the adversarial camouflage textures, we utilize a public dataset and a low-version YOLO model, specifically YOLOv3 [39]. This dataset is open-sourced by DAS, and the data samples are collected using CARLA [40]. The dataset comprises 12,500 training images and 3000 test images, accompanied by annotations including binary image masks, 3D vehicle models, and vehicle-camera position and pose parameters. The framework of our TACT is presented in Figure 3.

First, we redirect the classification supervision in both training and test sets to match the attack objective: all ground-truth vehicle labels are replaced with a predefined target class (e.g., “person”, “bird”). Subsequently, the 3D vehicle model (with predefined mesh topology) and initial random noise (serving as the texture seed) are jointly fed into a dedicated texture generator. The generator leverages the mesh face indices of the vehicle model to precisely locate the surface regions that need texture modification, synthesizes candidate adversarial camouflage textures, and maps them onto the corresponding surface of the 3D vehicle model. To bridge the gap between 3D texture mapping and 2D image presentation, a neural renderer is employed to render the textured 3D vehicle into a photorealistic 2D adversarial image, which is then seamlessly composited into the original scene via a transformation function. Finally, to optimize the adversarial texture, we directly adopt the original detector’s unmodified loss function and perform end-to-end optimization via backpropagation. Gradients are propagated backward through the transformation function, neural renderer, and texture generator, iteratively updating the texture until the detector consistently misclassifies the vehicle as the predefined target class.

We formulate the generation of the targeted adversarial camouflage texture $T_{\mathrm{adv}}^{\mathrm{tar}}$ as an optimization problem, where the texture is refined by minimizing a tailored loss function. The corresponding objective function is expressed as follows:

(4) $$\min_{T_{\mathrm{adv}}^{\mathrm{tar}}}\;L\left(T_{\mathrm{adv}}^{\mathrm{tar}}\right)=E_{\begin{array}{c} x∼X,\theta ∼\Theta \\ y_{t}∼Y \end{array}}\left[\ell \left(F_{\mathrm{full}}\left(T_{\mathrm{adv}}^{\mathrm{tar}},x,\theta \right),y_{t}\right)\right],$$

Here, $y_{t}∼Y$ denotes that the targeted attack class $y_{t}$ is sampled from the predefined set of target classes Y.

(5) $$F_{\mathrm{full}}\left(T_{\mathrm{adv}}^{\mathrm{tar}},x,\theta \right)=F\left(\Phi \left(R\left(M_{\mathrm{adv}}\left(T_{\mathrm{adv}}^{\mathrm{tar}}\right),\theta \right),m,x\right),\theta_{f}\right)$$

Our objective is to find the optimal targeted adversarial camouflage texture $T_{\mathrm{adv}}^{\mathrm{tar}}$ that minimizes the discrepancy between the output and the predefined target class $y_{t}$ over all original images, pose parameters, and target classes, thereby achieving a stable targeted adversarial effect for $T_{\mathrm{adv}}^{\mathrm{tar}}$. Algorithm 1 outlines the detailed steps of our TACT method.
**Algorithm 1** Targeted Adversarial Camouflage Texture Generation**Require:** Predefined target category, 3D mesh M, object detector *F*, training set (X,Y,Θ), maximum epochs **Ensure:** Targeted adversarial camouflage texture $T_{\mathrm{adv}}^{\mathrm{tar}}$
  1:**Supervision Redirection**: Redirect the classification supervision to the predefined target category  2:**for** i=1 **to** max_epochs **do**  3:       Sample an instance (x,y,θ) from the training set (X,Y,Θ)  4:       $I_{\mathrm{adv}}⇐R\left((M,T_{\mathrm{adv}}^{\mathrm{tar}}),\theta \right)$  5:       $x_{\mathrm{adv}}⇐\Phi \left(I_{\mathrm{adv}},m,x\right)$  6:       $O⇐F\left(x_{\mathrm{adv}};\theta_{f}\right)$  7:       Compute the adversarial loss $L_{\mathrm{adv}}$ via Equation (8)  8:       Optimize $T_{\mathrm{adv}}^{\mathrm{tar}}$ via Equation (4)  9:**end for**10:**return** $T_{\mathrm{adv}}^{\mathrm{tar}}$


#### 3.3.1. Gradient Redirection via Native Supervision Alignment

From a theoretical perspective, supervision redirection redefines the optimization signal for texture generation. Modern object detectors’ loss functions, such as YOLO’s $L_{\mathrm{cls}}$, are inherently effective feature aligners. In normal training, they force the model to map input images to the correct category feature space. Our supervision redirection strategy repurposes this inherent mechanism for adversarial ends.

Formally, the final adversarial image $x_{\mathrm{adv}}$ is a differentiable function of the texture parameters $\theta_{T}$. By redirecting the classification supervision to the target category $y_{t}$ in the classification loss $L_{\mathrm{cls}}=-\log p\left(y_{t}|x_{\mathrm{adv}};\theta_{f}\right)$, the gradient for texture optimization is computed via the chain rule:

(6) $$\nabla_{\theta_{T}}L_{\mathrm{cls}}=\underset{\mathrm{Altered}\;\mathrm{image}\;\mathrm{gradient}}{\underset{︸}{\left[-\nabla_{x_{\mathrm{adv}}}\log p\left(y_{t}|x_{\mathrm{adv}};\theta_{f}\right)\right]}}\cdot \underset{\mathrm{Gradient}\;\mathrm{through}\;\mathrm{pipeline}}{\underset{︸}{\nabla_{\theta_{T}}x_{\mathrm{adv}}\left(\theta_{T}\right)}}.$$

This shows the core mechanism: supervision redirection ($y\to y_{t}$) alters the primary image-space gradient $\nabla_{x_{\mathrm{adv}}}\log p\left(y_{t}|x_{\mathrm{adv}};\theta_{f}\right)$. Consequently, this repurposed signal guides $\theta_{T}$ to synthesize textures that increase the classifier’s belief in the target class $y_{t}$, achieving targeted feature alignment without modifying the loss function.

The sufficiency of redirected cross-entropy supervision is not unconditional. Our physical-domain experiments (Section 4.3) reveal that the formulation’s effectiveness depends on the semantic distance between the source class *y* and the target class $y_{t}$ in the detector’s feature space: when $y_{t}$ relies on fine-grained, high-frequency features that are fragile under the physical pipeline (e.g., the plumage textures discriminative of the “bird” class), the targeted alignment partially collapses and the attack degrades into a non-targeted disruption. We discuss this phenomenon in detail in Section 4.3 and Section 5, and view it as a direct empirical signature of the information-theoretic limits of the redirected supervision: the cross-entropy symmetry guarantees a valid optimization direction, but the physical realizability of the resulting texture is bounded by the information capacity of the printing–capture channel.

#### 3.3.2. Adversarial Loss

The adversarial loss inherits the original detector’s loss structure, theoretically minimizing the discrepancy between model output and the predefined target category feature distribution:

(7) $$L_{\mathrm{total}}=\alpha L_{\mathrm{box}}+\beta L_{\mathrm{obj}}+\gamma L_{\mathrm{cls}}$$

Our attack targets the classification branch of the detection model, and the adversarial loss retains the original loss structure without any modification. While the definition of each loss term remains unchanged, the adversarial property of the texture is mainly reflected in the targeted optimization of the classification loss, guiding the generated texture to align with the feature distribution of the predefined target category. The overall adversarial loss is defined as:

(8) $$L_{\mathrm{adv}}=\alpha L_{\mathrm{box}}+\beta L_{\mathrm{obj}}+\gamma L_{\mathrm{adv}}^{\mathrm{cls}}\;$$

Here, α, β, and γ denote the weights of each loss term in $L_{\mathrm{adv}}$, which are used to balance the contribution of individual loss terms. These weights are set identically to those in the original loss function in Equation (7).

The final adversarial camouflage texture obtained by the model after training is the pattern that can make the detection model recognize it as the predefined target category. During inference, the object detector extracts features consistent with the target category from the adversarial texture, rather than the intrinsic features of the original target, thereby achieving targeted adversarial effects. Notably, TACT only modifies the surface texture of the target vehicle; the vehicle’s intrinsic attributes—including geometric shape, material composition, and mechanical performance—remain entirely unchanged throughout this process.

### 3.4. Theoretical Connection

#### 3.4.1. Feature Adversarial Attack Theory

TACT is grounded in the theory of feature-space adversarial attacks. As demonstrated by ref. [41], adversarial attacks can operate directly at the feature level by minimizing the distance to target features. The native supervision redirection mechanism of TACT essentially leverages the geometric structure of the feature space learned by the detector during pre-training: by aligning the optimization direction with the target category $y_{t}$, we force the optimization process to search for texture parameters such that the feature representation $\phi (x_{\mathrm{adv}})$ of the rendered image approximates the feature prototype $\mu_{y_{t}}$ of category $y_{t}$ internal to the detector. This can be formalized as:

(9) $$\min_{\theta_{T}}D\left(\phi \left(x_{\mathrm{adv}}\left(\theta_{T}\right)\right),\mu_{y_{t}}\right)$$

where *D* denotes a distance metric in the feature space. This perspective is consistent with TACT’s observed cross-model transferability: different detectors pre-trained on the same dataset may learn similar feature space structures.

#### 3.4.2. Feature Transferability Theory

The cross-model transferability of TACT can be explained by the theory of feature transferability in deep neural networks (DNNs). Ref. [42] experimentally demonstrated that the generic features learned in the early layers of DNNs are highly transferable across different tasks and architectures. Although the detector used for our white-box optimization and the black-box target models differ in architecture and training details, both are pre-trained on the COCO dataset and thus share similar low- and mid-level feature representations. Consequently, to the extent that adversarial perturbations operate in these shared feature subspaces, the adversarial textures optimized on the surrogate model can effectively transfer to other detectors. This reveals a key property of physical adversarial attacks: transferability depends more on inter-model feature representation similarity than on alignment of decision boundaries.

## 4. Experiments and Results

We evaluate the effectiveness, robustness, and transferability of our TACT method in both simulated and real-world scenarios. Our experimental analysis encompasses four key components: adversarial effectiveness experiments, targeted robustness tests, transfer-based black-box experiments, and ablation studies on the smooth loss term.

### 4.1. Experimental Settings

#### 4.1.1. Implementation Details

The TACT method is implemented based on the PyTorch 1.8 framework, and the training of adversarial camouflage is conducted on a single NVIDIA RTX 3090 GPU. Instead of collecting a dedicated dataset and performing weight training from scratch, we directly adopt the pre-trained weights of public datasets to validate the effectiveness of our method, which is particularly resource-efficient for researchers with limited computational power.

We adopt YOLOv3 as the surrogate model to optimize the adversarial texture in a white-box manner. We then conduct black-box transfer experiments to verify its cross-model effectiveness on unseen mainstream detectors, including higher-version YOLO detectors (YOLOv5, YOLOv8, and YOLO11) and detectors from other frameworks such as Faster R-CNN [43], Mask R-CNN [44], SSD [45], and Real-Time Detection Transformer (RT-DETR) [46]. All these models are pre-trained on the MS COCO dataset, enabling them to identify and localize 80 categories of common objects. In our experiments, YOLOv5, YOLOv8, YOLO11, and RT-DETR are sourced from the Ultralytics library [47], while Faster Regions with Convolutional Neural Networks Features (Faster R-CNN), Mask R-CNN, and SSD are provided by the MMdetection library [48]. Since our work targets general-purpose detectors, we apply no modifications to their architectures or pre-trained weights, thereby preserving their inherent robustness and generalization capability.

The surrogate model used during training is YOLOv3, with architecture and weights identical to those of the original implementation. All hyperparameters and threshold settings strictly follow the configurations in the original implementation. The initial texture is set to random Gaussian noise. The adversarial camouflage textures are trained for 5 epochs using the Adam optimizer with an initial learning rate of $10^{-2}$ and a batch size of 1. The texture size is set to 6 pixels and initialized with uniform random noise in [0,1). No data augmentation is applied. The neural renderer uses the soft rasterizer with a fixed camera model as described in the original DAS framework [7]. Training requires approximately 2.5 h per epoch on a single RTX 3090 GPU.

#### 4.1.2. Evaluation Metrics

We evaluate TACT and baseline methods using four metrics: Average Precision at IoU threshold 0.5 (P@0.5), Miss Rate (MR), Targeted Attack Success Rate (TASR), and Targeted Classification Error Rate (TCER). The latter two metrics are specifically designed for targeted physical attack evaluation and formally defined below. For targeted attacks, a successful targeted attack inherently implies a successful attack (in the general sense), but a successful general attack does not guarantee a successful targeted attack. Since non-targeted attacks are not the focus of our method, TASR and TCER differ in both numerator and denominator: TASR takes the number of samples with correct localization and misclassification into the predefined target category as its numerator and the total number of correctly localized samples as its denominator, while TCER uses the sum of the aforementioned misclassified samples and missed detection samples as its numerator and the total number of test samples as its denominator.

P@0.5 reflects the model’s precision when the IoU threshold is set to 0.5, and its calculation formula is:

(10) $$P@0.5=\frac{TP}{TP+{FP}^{\prime }}$$

where TP (True Positives) denotes the number of correctly detected targets, and FP (False Positives) denotes the number of incorrectly detected targets.

Recall rate in object detection tasks refers to the proportion of all ground-truth bounding boxes that are correctly detected by the model in both position and category, and is expressed by the formula:

(11) $$R=\frac{TP}{TP+{FN}^{\prime }}$$

The Miss Rate (MR) is an indicator that measures the probability of a model failing to identify true targets, and it is complementary to the recall rate. Its definition is as follows: it refers to the proportion of all ground-truth bounding boxes that are not correctly detected by the model, including cases of mismatched positions, misclassification, or complete failure of detection.

(12) $$MR=1-R=\frac{FN}{TP+{FN}^{\prime }}$$

where FN (False Negatives) denotes the number of ground-truth bounding boxes that fail to be correctly detected by the model.

TASR is a dedicated metric tailored for evaluating targeted adversarial attacks in object detection, which is distinct from the Targeted Attack Success Rate used in image classification tasks [49,50,51]. It only focuses on samples that are correctly localized by the detector but misclassified into the predefined target category. Specifically, TASR is calculated as the count of such misclassified samples divided by the total number of samples correctly localized by the detector, with the formula expressed as:

(13) $$\mathrm{TASR}=\frac{N_{\mathrm{tar-mis}}}{N_{\mathrm{loc-corr}}}\times 100\%,$$

here, $N_{\mathrm{tar-mis}}$ denotes the number of test samples that are correctly localized by the detector and misclassified into the predefined target incorrect category (the intended category for the adversarial attack), while $N_{\mathrm{loc-corr}}$ represents the total number of samples in the test set that are correctly localized by the detector.

TCER is a dedicated metric for evaluating targeted adversarial attacks, which focuses on samples where the detector either correctly localizes the object but misclassifies it into the predefined target incorrect category or completely fails to detect the target. Specifically, TCER is calculated as the sum of the count of such misclassified samples and missed detection samples divided by the total number of test samples, with the formula expressed as:

(14) $$\mathrm{TCER}=\frac{N_{\mathrm{tar-mis}}+N_{\mathrm{miss}}}{N_{\mathrm{test}}}\times 100\%,$$

here, $N_{\mathrm{miss}}$ represents the number of test samples where the detector completely fails to detect the real target, while $N_{\mathrm{test}}$ represents the total number of samples in the test set.

### 4.2. Evaluation in Simulation Settings

#### 4.2.1. Baseline Methods

We evaluate the performance of our proposed TACT method on the test set and further conduct a comparative analysis against current camouflage methods, including several publicly available approaches for generating adversarial camouflage textures, raw textures, and textures constructed from Gaussian random noise. The baseline methods involved in the comparison include CAMOU [30], ER [31], FCA [11], DAS [7], ACTIVE [9], MFA [32], DTA [12], MCC [13], and RAUCA [14].

**Figure 4:**
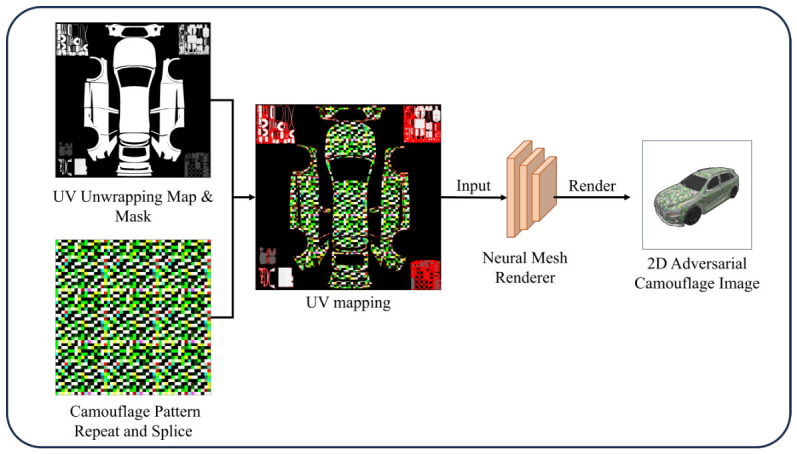
Pipline of converting the UV image into a texture file and rendering it as a 2D adversarial camouflage image. Taking CAMOU as an example.

For a fair comparison, all baseline textures are mapped onto the same 3D vehicle model and rendered into 2D images using the same neural renderer. A physical transformation functionΦ is then applied to bridge the gap between the digital simulation and physical deployment domains. Specifically, for methods that have publicly released their texture mapping relationships (e.g., FCA, DAS, MFA, RAUCA), we directly adopt their provided textures for image rendering and detection experiments. For methods that have only disclosed texture patterns without releasing the texture mapping relationships (e.g., ER, CAMOU, DTA, ACTIVE), we convert the texture patterns reported in their papers into UV maps for the target vehicle model, and then map these UV maps to texture files by combining them with the model’s surface index files, as shown in Figure 4. For MCC, which also targets targeted attacks, direct quantitative comparison is infeasible as its code and trained models are not publicly available. To facilitate a fair comparison, we have reproduced a texture by strictly adhering to the methodological descriptions provided in its original paper. These generated texture files are subsequently used for rendering and test sample preparation prior to conducting detection experiments.

**Figure 5:**
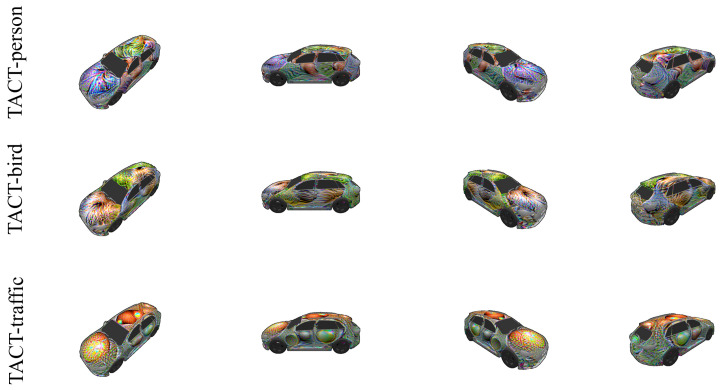
Visualization of TACT-based adversarial camouflage from different perspectives.

Three types of targeted adversarial camouflage are included in our comparative experiments, namely those designed to induce misclassification into the “person”, “bird”, and “traffic light” categories. The three types of adversarial camouflage generated by TACT are illustrated in Figure 5.

#### 4.2.2. Attack Effectiveness

**Figure 6:**
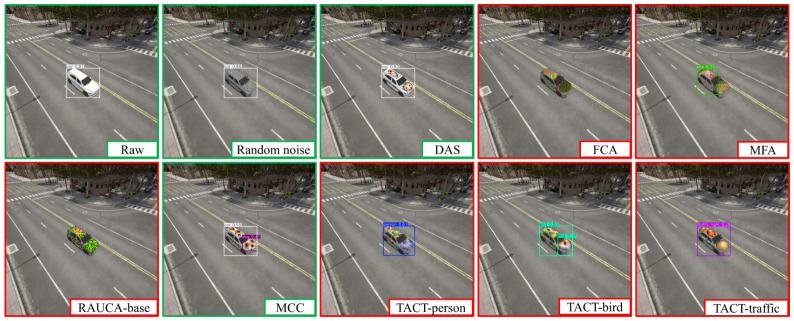
Visualization of detection results of different adversarial camouflages on YOLOv5.

**Figure 7:**
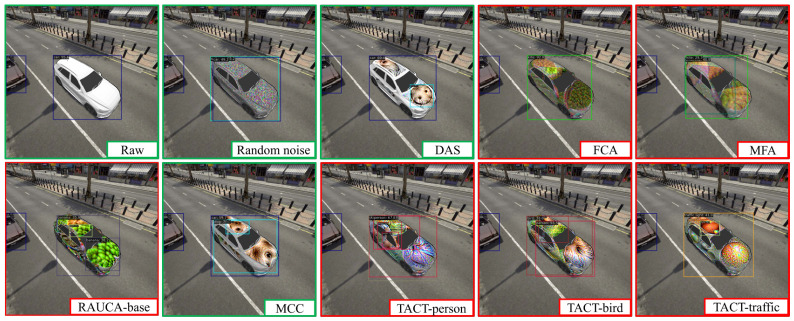
Visualization of detection results of different adversarial camouflages on SSD.

We conduct extensive attack comparisons between TACT and ten adversarial camouflage baselines across seven mainstream detectors. Table 2 presents the P@0.5 (%) and MR (%) metrics across all detectors, while Figure 6 and Figure 7 visualize the detection results under different adversarial camouflage strategies. The most effective methods are highlighted through color-coding. Specifically, red denotes the optimal adversarial camouflage for a detector, orange the suboptimal, cyan the third optimal, green the fourth optimal, and gray the fifth optimal.

Figure 6 and Figure 7 illustrate the attack effectiveness of different adversarial camouflages against different object detectors; the green border indicates detection is correct (attack failed), and the red border indicates detection is incorrect (attack successful). All TACT-derived adversarial camouflage configurations achieve successful attacks and enable effective targeted attacks.

The results in Table 2 indicate that RAUCA achieves the overall optimal attack performance across most object detectors, while FCA obtains competitive suboptimal results, especially on the YOLO series detectors. MFA disrupts critical multi-layer object-aware features at the pixel level, achieving suboptimal results on most detectors and ranking among the top three across all evaluated models. The performance of DAS is inferior to that of full-coverage random noise on some detectors. Although DAS suppresses attention from both models and humans simultaneously, its partial-coverage patch-based design limits its overall attack effectiveness. As for MCC, it also exhibits favorable attack performance, though not as good as RAUCA, FCA, MFA, and the proposed TACT. Nevertheless, MCC can achieve targeted adversarial attacks from multiple angles, which may, to a certain extent, compromise its other attack capabilities, such as bounding box misalignment or confidence reduction. Unlike existing state-of-the-art methods that are predominantly non-targeted, TACT supports controllable targeted generation toward user-specified categories, with effectiveness systematically validated across seven general-purpose object detectors. Among all TACT variants, TACT-bird is the top performer: it reduces the P@0.5 score of SSD by 67.9%, and elevates the corresponding MR by 54.4%. TACT-person ranks second among all TACT camouflage methods, consistently delivering stable and competitive attack results across multiple detectors. It boosts the MR of Faster R-CNN, SSD, and RT-DETR above 80%, and elevates the MR of YOLOv5 and Mask R-CNN beyond 70%. These results demonstrate that TACT not only achieves attack effectiveness and cross-architecture transferability comparable to SOTA non-targeted methods, but also enables flexible targeted adversarial control, with stable performance against both upgraded YOLO variants and other mainstream detection architectures.

#### 4.2.3. Attack Targetedness

Given that only the MCC is designed for targeted attacks, we restrict the TASR and TCER comparison to the three TACT variants and those generated by the MCC method, as shown in Table 3 and Table 4. The global confidence threshold is set at 0.25. We ignore objects in the background that are detected as specified classes in our statistics, and only count the number of times the target object is misclassified as a specified incorrect class. Even if the same target is detected as two specified class objects in an image, we only count once.

In terms of targeted attack performance evaluated by TASR and TCER, the proposed TACT variants achieve substantial improvements over the baseline MCC. In terms of average TASR, TACT-person reaches 51.91%, and TACT-traffic achieves 50.10%, both remarkably outperforming MCC (37.77%) and TACT-bird (32.95%). In terms of average TCER, TACT-person and TACT-traffic also attain competitive results of 58.63% and 58.18%, respectively, which are far higher than MCC (45.19%) and TACT-bird (46.36%). Overall, TACT-person and TACT-traffic deliver the best comprehensive targeted attack capability across all detectors. TACT-bird exhibits superior performance only on YOLOv5 and YOLOv8, while its results degrade sharply on cross-architecture detectors and reach the lowest TASR and TCER on Mask R-CNN. By comparison, TACT-traffic shows more consistent performance across architectures and achieves particularly strong targeted attack results on RT-DETR. The baseline MCC presents relatively uniform performance across detectors but is obviously inferior to TACT-person and TACT-traffic in overall targeted attack ability. It is also observed that all camouflage methods yield excellent targeted attack results on the SSD detector with high TASR and TCER values.

**Figure 8:**
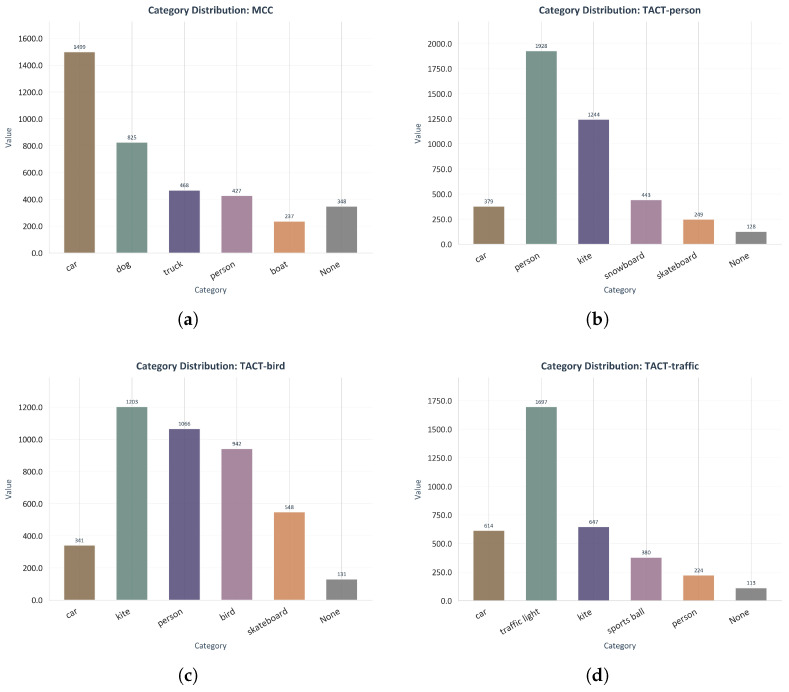
Top-5 category detection counts and undetected object counts across different adversarial camouflage. (**a**) MCC; (**b**) TACT-person; (**c**) TACT-bird; (**d**) TACT-traffic.

These results demonstrate that the proposed TACT variants exhibit cross-architecture transferability, enabling effective targeted misclassification against a wide range of mainstream object detectors. Figure 8 further statistically analyzes the top-5 detected targets and undetected outputs under different camouflage strategies with Faster R-CNN. The detector produces the highest detection count of person-class targets under TACT-person, and bird-class targets rank third under TACT-bird. Meanwhile, partial attack failure still exists: vehicle targets are always among the top-5 detection results across all camouflage settings, and undetected targets also occupy a considerable proportion in the statistics.

### 4.3. Evaluation in Real-World Settings

In this section, we evaluate the effectiveness of our proposed TACT in real-world settings. Based on the digital-domain results, we select four adversarial camouflage textures (FCA, RAUCA, TACT-person, and TACT-bird), along with raw vehicle texture as a clean reference, for physical-domain experiments.

**Figure 9:**
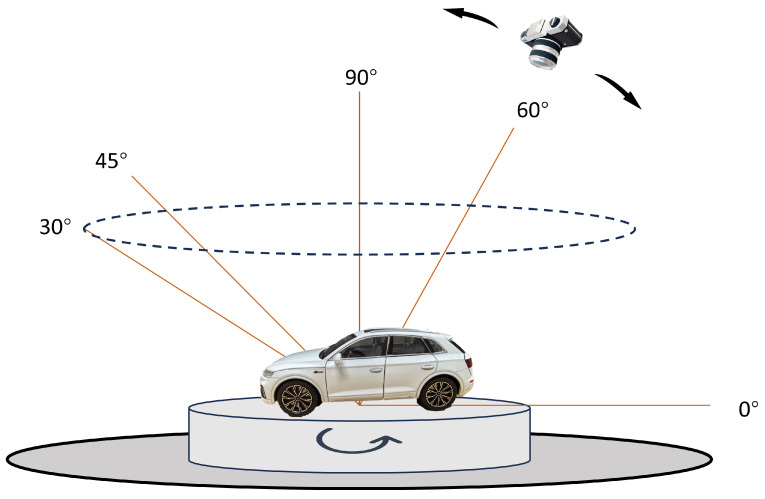
Experimental setup for collecting test sets in the physical domain.

To replicate adversarial camouflage and construct the real-world test dataset, we employed the following procedure: First, adversarial camouflage textures were printed on polypropylene poster material using a large-format inkjet printer. These printed textures were then cut to size and affixed to 1:24 scale model vehicles, simulating the appearance of automotive coatings in real-world scenarios. The model vehicles with different adversarial camouflage textures were placed on a rotating display platform with a rotational speed of approximately 3.16 r/min. Video recordings were captured at a distance of 1 m from the models, at five angular positions: 0°, 30°, 45°, 60°, and 90°. Recording was paused after each full rotation of the platform. Images were extracted from the video footage at intervals of 10 frames. A total of 2869 images were collected. The experimental setup is illustrated in Figure 9.

**Figure 10:**
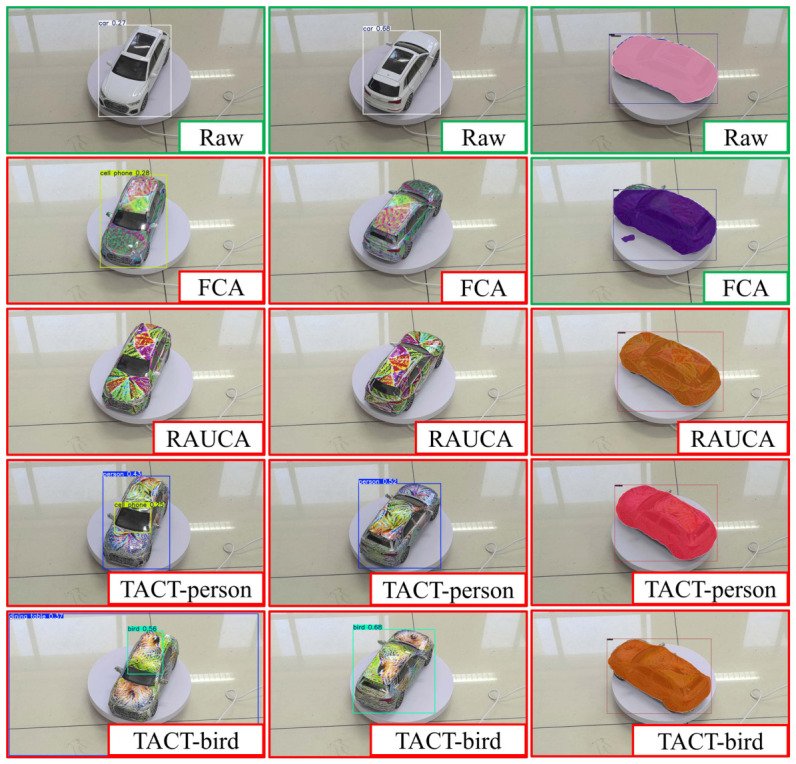
The attack effects of different adversarial camouflages in the real world. Red indicates a successful attack (i.e., detection failure), and green indicates a failed attack (i.e., detection success). TACT can successfully attack object detectors and mislead them into identifying pre-specified categories.

We evaluated the attack efficacy of various adversarial camouflages under laboratory conditions. Raw texture and those with adversarial camouflages were tested on different detectors, with detection results captured from different shooting angles and the comprehensive detection results across all angles recorded separately, as presented in Table 5. We use bold type to indicate the optimal attack effect and underlining to denote the suboptimal attack effect. Mean Average Precision from IoU 0.5 to 0.95 (mAP50-95) is a comprehensive evaluation metric that averages precision across IoU thresholds of 0.5, 0.55, …, 0.95, providing a more rigorous assessment than single-threshold metrics. TACT-bird achieved optimal attack performance on all detectors, with a 7.18% improvement in attack performance on mAP50-95 on YOLOv8 compared with state-of-the-art adversarial camouflage RAUCA. Furthermore, the attack performance of adversarial camouflage becomes increasingly stronger as the shooting angle rises; at 60° and 90°, RAUCA, TACT-bird, and TACT-person can almost fully induce misjudgment in the YOLOv8 detector. This indicates that the more obvious the features such as wheels and windows, the higher the probability of the object detector identifying the object correctly; in contrast, the higher the shooting angle is, the better the attack effect of full-coverage adversarial camouflage will be. Figure 10 illustrates the physical-domain attack performance of TACT, where red bounding boxes indicate successful attacks (missed detections or misclassifications) and green bounding boxes indicate attack failures (correct detections).

Table 6 presents the quantitative results of targeted attacks for TACT in the physical domain. When using a Mask R-CNN detector for detection, TACT-person exhibits excellent performance: at a shooting angle of 90°, the TASR can reach 88.60%. Under YOLO-based detectors, the attack primarily induces missed detections rather than targeted misclassification, as reflected by the low TASR but high TCER values in Table 6. Moreover, both textures show a trend of increasing targeted attack effects as the shooting angle increases.

It is noteworthy that while TACT-bird achieves the strongest overall attack performance in the physical domain (achieving the lowest mAP50-95 across all detectors, as shown in Table 5), its physical-domain TASR is substantially lower than that of TACT-person. This discrepancy is consistent with an empirical pattern observed across target categories: bird recognition is known to rely on fine-grained, high-frequency texture patterns (e.g., plumage detail, beak contour) [52] that are more susceptible to degradation through the physical printing–capture pipeline than the coarse-grained body shape and color distribution features characteristic of person-class recognition. Furthermore, the large semantic distance between the source class (car) and the target class (bird) in the detector’s feature space requires the adversarial texture to encode a more extreme feature perturbation, resulting in a fragile feature configuration that is more easily disrupted by physical-domain noise. This interpretation is supported by the TCER–TASR gap observed for TACT-bird on YOLOv5: the TCER (47.99%) substantially exceeds the TASR (4.81%), indicating that the texture successfully induces detection failure (missed detections) but fails to steer the detector’s prediction toward the bird class. In other words, the non-targeted disruption component of TACT-bird survives physical deployment, but the targeted feature alignment to the bird prototype does not; the texture successfully induces detection failure, yet it cannot reliably guide the detector’s prediction toward the bird class. This pattern indicates that high-frequency discriminative features are more vulnerable to physical-domain degradation than low-frequency ones.

### 4.4. Ablation Studies

In this section, we conduct ablation experiments to explore the influence of the core supervision redirection mechanism on the performance of adversarial camouflage. The proposed supervision redirection mechanism reshapes the optimization supervision signal to drive adversarial textures toward the feature distribution of predefined target categories, as detailed in Section 3.3.1. To verify its necessity, we construct a baseline variant TACT-car that adheres to the original category supervision setting, and compare it with uncamouflaged raw vehicles in terms of Recall and mAP50-90.

Table 7 shows TACT-car outperforms Raw in Recall on 6 out of 7 detectors, e.g., by 8.85 percentage points on YOLOv5, and improves mAP50-90 for YOLO variants. Only RT-DETR shows degradation, likely due to its strong feature learning capability. This confirms TACT-car strengthens alignment with “car” features, making vehicles more detectable. Thus, the supervision redirection mechanism is indispensable for TACT: without this mechanism, the generated textures will only enhance the feature representation of the original vehicle category rather than mimicking the features of the predefined target categories, ultimately failing to achieve targeted adversarial misclassification.

### 4.5. Mechanism Validation via Class Activation Mapping

In this section, we leverage Class Activation Mapping (CAM) to visualize the model’s attention distribution under TACT, providing visual support for the supervision redirection hypothesis.

To analyze TACT’s impact on the feature perception and decision-making of object detectors, we generate CAM visualizations [53] using multi-scale feature maps extracted during model inference. Specifically, we visualize attention distributions for three class categories: person (index = 0), car (index = 2), and bird (index = 14), and qualitatively examine the model’s high-response feature regions under clean and adversarial inputs.

**Figure 11:**
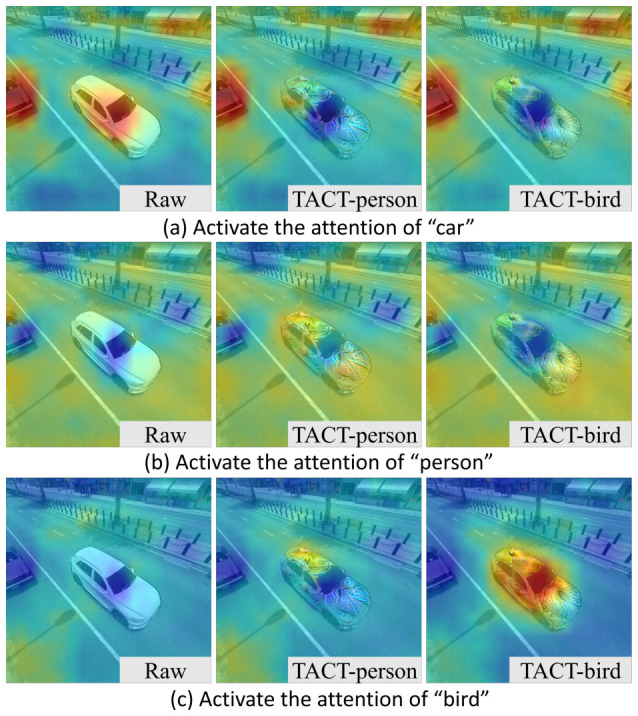
Visualization results of CAM for YOLOv5 of different classes.

The visualization results use color gradient to represent the feature response intensity of the model: the red/bright yellow areas are the high response areas, indicating that the model pays more attention to the features of that location, which is the core basis for the model to identify the target; The blue/cyan area is a low response area, representing the area determined by the model as the background. The CAM results are shown in Figure 11. When the “bird” and “person” classes are activated, the model’s attention is clearly focused on the corresponding TACT adversarial camouflage vehicles; when the “car” category is activated, the model’s attention concentrates on the raw vehicle, while attention on the TACT-camouflaged vehicle is strongly suppressed.

### 4.6. Low-Pass Filtering Analysis

The printing–capture pipeline inherently acts as a low-pass filter, attenuating high-frequency components through optical blurring and sampling loss. To characterize this degradation, we apply Gaussian blur (σ∈{0,1,2,3,5,7,10,15}) to the rendered textures before feeding them to the YOLOv3 detector, using foreground-only texture variants (annotation region only, background removed) to isolate the effect on texture content.

**Figure 12:**
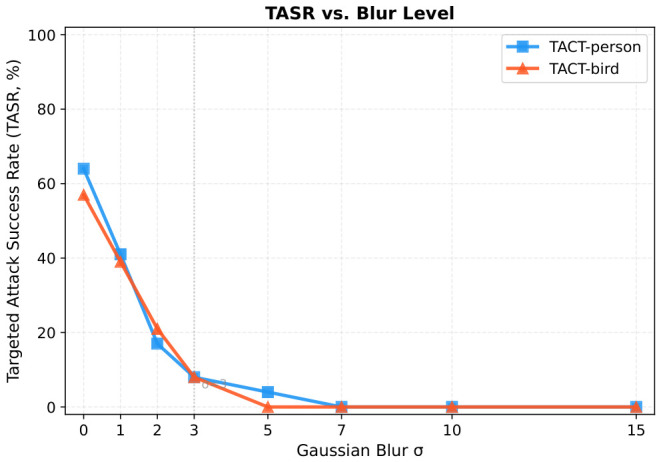
TASR vs. Gaussian blur sigma for TACT-person and TACT-bird textures. Both variants degrade sharply under low-pass filtering.

Figure 12 shows the TASR as a function of blur strength. Both TACT-person and TACT-bird degrade sharply: from 64% and 57%, respectively, at σ=0 to below 10% at σ=3, confirming that the simulated low-pass filtering effect causes a steep information loss across all texture types. This steep degradation is consistent with the physical-domain results, where both textures show substantially reduced mAP50-95 compared with their digital-domain performance, supporting the premise that the printing–capture pipeline imposes a strong low-pass filtering effect.

### 4.7. Robustness to Environmental Variations

**Figure 13:**
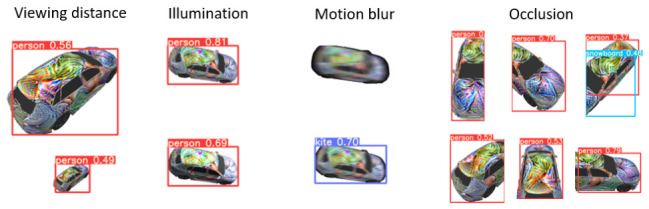
Detection results of TACT-person under varying environmental conditions.

To provide a preliminary assessment of TACT’s robustness under varying conditions, we conduct four simulation experiments using the existing differentiable rendering pipeline: illumination variation, viewing distance variation, motion blur, and partial occlusion, all with the TACT-person texture evaluated on YOLOv3. TACT-person maintains its targeted misclassification effect under illumination changes, different viewing distances, and partial occlusion, suggesting reasonable robustness to these common environmental factors. Motion blur presents a more informative case: under simulated motion blur, targeted attack fails, but non-targeted disruption (misclassification and missed detection) remains effective. Figure 13 shows the effect of robustness testing.

### 4.8. Extended Experiments

**Figure 14:**
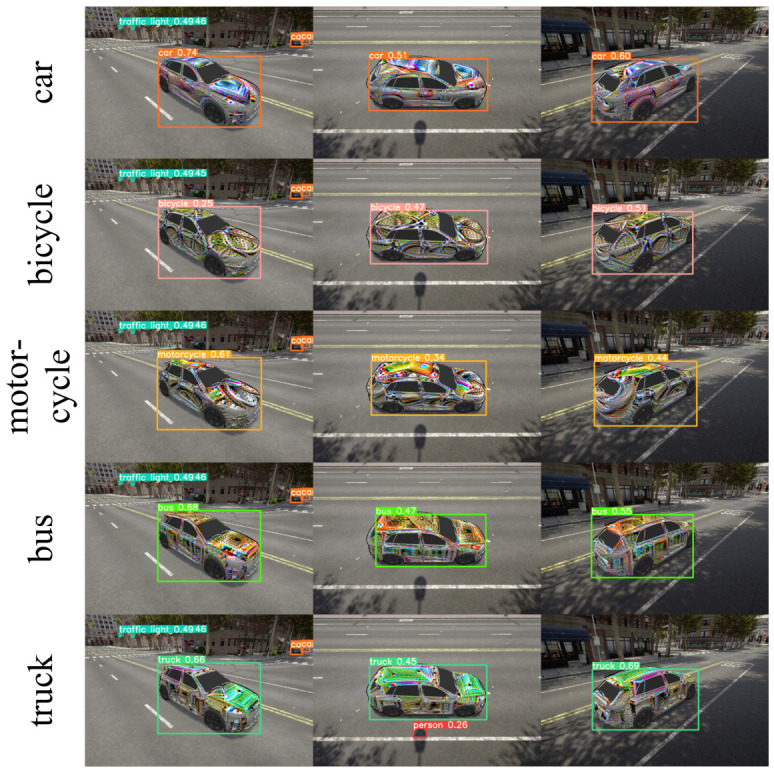
Comparison and detection results of adversarial examples generated by TACT with similarity characteristics in the digital domain. Detected by YOLOv3.

In this section, we conduct extended experiments on the proposed method. Specifically, we select real-world target classes that share similar visual features with “car” and explore whether objects with similar real-world features are also cognitively similar in the model’s perception. We selected several wheeled objects with feature similarities to “car”—namely “bicycle”, “motorcycle”, “bus”, and “truck”—and designated them as the target classes for supervision redirection. These target classes are chosen based on their structural and visual similarities to “car”, ensuring the experiment can effectively verify the generalization ability of the proposed method across similar object categories. The comparison and detection results of adversarial examples in the digital domain are illustrated in Figure 14.

It can be observed that several types of adversarial camouflage can induce the model to classify the target into the specified class. Furthermore, even though these classes share similar real-world features with the “car” class. For instance, “truck” and “bus” are both equipped with wheels and windows, while “bicycle” and “motorcycle” both have two wheels, and even though models often misclassify one of these classes as another in general object detection tasks (e.g., frequently detecting a “car” as a “bus” or a “truck”). This observation suggests that the model’s latent feature space does not fully mirror human perceptual similarity, representing a promising direction for future investigation.

## 5. Discussion

Building on our experimental results, this section examines the mechanistic basis of TACT’s effectiveness, characterizes the conditions influencing the physical-domain effectiveness of the native supervision redirection strategy, and situates our findings within the broader context of physical adversarial attack research. We find that the detector’s native, unmodified loss function is sufficient to drive full-coverage 3D adversarial texture optimization toward arbitrary target categories—a capability that prior physical adversarial camouflage methods, including non-targeted approaches, did not exploit.

### 5.1. Mechanism and Implications of Native Loss

During pre-training on large-scale datasets such as MS COCO, the cross-entropy loss forces the detector to construct a feature space in which each category occupies a distinct, well-separated region. This feature space is not a side effect of training—it is the training objective. By substituting the supervision label from *y* to $y_{t}$, TACT inverts this mechanism: the redirected gradient encourages the texture parameters $\theta_{T}$ to align with features associated with the target category. The CAM visualizations in Section 4.5 provide qualitative support for this interpretation: under TACT-person and TACT-bird textures, the detector’s high-response regions shift from the vehicle’s structural features (wheels, windows) to the texture surface itself, which is consistent with the hypothesis that the native loss has redirected feature attention toward the target class.

This mechanism has a non-obvious implication: it means that the complexity introduced by prior methods—custom smooth losses, stealth losses, attention suppression losses—addresses problems other than targeted feature alignment. Those components serve legitimate purposes (improving visual naturalness, enhancing transferability, handling environmental variation), but they are orthogonal to the core targeted misclassification objective. TACT demonstrates that these two concerns can be decoupled: targeted feature alignment requires only label substitution; auxiliary components can be added independently if naturalness or robustness is additionally required. This decoupling simplifies the design space for future targeted physical attacks and provides a cleaner baseline against which more complex methods can be evaluated.

From a security perspective, this finding carries a sobering implication. Targeted physical adversarial attacks against deployed detection systems do not require specialized knowledge of loss function design or access to auxiliary training infrastructure. An attacker with access to a pre-trained detector and a differentiable rendering pipeline can mount effective targeted attacks using only the detector’s public training objective. This low barrier to entry suggests that the threat surface of targeted physical adversarial attacks is broader than previously appreciated, and underscores the need for detection-side robustness measures that are specifically evaluated against targeted, category-controlled attacks.

### 5.2. Empirical Pattern of Physical-Domain Effectiveness

The sufficiency of native supervision redirection is not unconditional. Our physical-domain results reveal an empirical pattern: this strategy’s effectiveness depends on the visual properties of the target class as represented in the detector’s feature space.

For target classes with coarse-grained and low-frequency discriminative features such as the body shape and color distribution of the person category, the redirected gradient synthesizes textures whose category-relevant features can survive the physical printing–capture pipeline with high fidelity. TACT-person achieves 88.60% TASR at 90° on Mask R-CNN and maintains competitive TASR across multi-viewing angles, confirming that person-class features are robustly transferable through the physical channel.

For target classes with fine-grained and high-frequency discriminative features, including the plumage detail and beak contour of the bird category, the situation is fundamentally different. Although TACT-bird achieves strong targeted alignment in the digital domain (TASR of 66.29% on YOLOv8), its physical-domain TASR collapses to 4.81% on YOLOv5, while its TCER remains at 47.99%. This TCER–TASR gap is diagnostic: the texture successfully disrupts detection (the non-targeted component survives physical deployment) but fails to steer the detector’s prediction toward the bird class (the targeted component does not). This decoupling has two practical implications. First, it simplifies the design space: targeted feature alignment requires only label substitution, and auxiliary components for naturalness or robustness can be added independently as needed. Second, and more importantly, it reframes how we evaluate progress in this area. If a future method achieves higher TASR than TACT, the gain must come from components beyond label substitution—and those components can now be ablated cleanly against our baseline. TACT thus serves not only as an attack method but as a minimal reference point that isolates the contribution of any additional complexity introduced by more sophisticated approaches.

This empirical pattern suggests that the physical-domain viability of native supervision redirection consistently varies with the spatial frequency requirements of the target class: categories with discriminative features below the pipeline’s effective frequency cutoff achieve reliable physical-domain attack success, while those above it do not—at least without additional mechanisms to preserve high-frequency texture fidelity. Target class selection is therefore not a neutral design choice in physical-domain targeted attacks, and practitioners should consider target class feature granularity when evaluating worst-case security vulnerabilities.

### 5.3. Limitations and Future Directions

Physical robustness. TACT exhibits reduced attack effectiveness at low viewing angles (≤30°), where structural vehicle features (wheels, windows) dominate the detector’s feature response and partially override the adversarial texture signal. This is consistent with the mechanism described in Section 5.1: at angles where geometry-based features are prominent, the texture-based targeted alignment must compete against stronger structural priors. Future work could integrate viewing-angle diversity and lighting variation directly into the texture optimization objective, following the multi-weather augmentation approach of RAUCA [14].

Defense awareness. TACT-generated textures are visually conspicuous, making them potentially detectable by defense methods such as adversarial training, input transformations, or feature-squeezing-based detectors. Since TACT is not designed to be stealthy at the image level, its adversarial textures are likely to be identified as anomalous by statistical detection methods that analyze local image statistics or frequency-domain characteristics. Future work could explore making TACT textures more resilient to such defenses, for instance by incorporating perceptual constraints that reduce visual distinctiveness without compromising the core supervision redirection mechanism.

Visual naturalness. TACT-generated textures are visually conspicuous, limiting their utility in scenarios where stealth is required. Since our results establish that targeted feature alignment and visual naturalness are decoupled objectives (Section 5.1), a dual-objective framework that jointly optimizes adversarial effectiveness and perceptual naturalness—for instance via neural style transfer [54]—is a natural extension that does not require modifying the core supervision redirection mechanism, but naturalness constraints can be incorporated as additive regularization terms, at the cost of a performance–naturalness trade-off governed by the relative loss weights.

Evaluation scale. The physical experiments in this work follow the scale-model evaluation protocol widely adopted in the adversarial camouflage literature [11,14], which enables controlled multi-angle assessment while preserving the key physical variables of the printing–capture pipeline. However, scale models differ from full-size vehicles in surface area, viewing distance, and background complexity, all of which may affect the physical-domain TASR. Extending evaluation to full-scale vehicles in uncontrolled outdoor environments remains an important open problem, particularly for assessing whether the consistent trend observed between target class feature granularity and physical-domain attack effectiveness generalizes beyond the scale-model setting examined in this work.

## 6. Conclusions

This work demonstrates that targeted physical adversarial camouflage does not require the complex loss function designs adopted by prior methods. By redirecting the classification supervision label toward a predefined target category without redesigning the detector’s loss function, introducing auxiliary networks, or modifying the rendering pipeline, TACT achieves targeted attack success rates competitive with or exceeding substantially more complex methods across seven detectors and two domains. This finding challenges a prevailing implicit assumption in the physical adversarial camouflage literature: targeted misclassification requires non-trivial optimization machinery.

Beyond this core finding, we introduce TASR and TCER as dedicated evaluation metrics for targeted physical attacks, and reveal a trend: coarse-grained target categories (e.g., person) consistently achieve higher physical-domain TASR than fine-grained ones (e.g., bird). Through low-pass filtering simulation and weight-space projection analysis, we provide convergent evidence that this asymmetry arises from two complementary factors: (1) the printing–capture pipeline acts as a strong low-pass filter that attenuates texture information across all target classes, and (2) rendered textures align with target class weight vectors in the detector’s feature space, with the strength and stability of this alignment correlating with physical-domain robustness. These results suggest that the threat surface of targeted physical adversarial attacks is both broader and more structured than previously appreciated. This work provides a new perspective for the design of targeted adversarial camouflage, offers a reference for defensive strategy development, and can serve as a baseline for evaluating detector vulnerability to targeted physical attacks.

## Figures and Tables

**Table 1:** Comparison of physical adversarial camouflage.

Method	Years	Differentiable	Targeted	Full-Coverage	Ref.
CAMOU	2019	×	×	✓	[30]
ER	2020	×	×	✓	[31]
DAS	2021	✓	×	×	[7]
FCA	2022	✓	×	✓	[11]
DTA	2022	✓	×	✓	[12]
ACTIVE	2023	✓	×	✓	[9]
MCC	2024	✓	✓	×	[13]
RAUCA	2025	✓	×	✓	[14]
TACT(ours)	2026	✓	✓	✓	/

Notes: ✓ indicates that the method possesses the corresponding property, and × indicates that the method does not possess the corresponding property.

**Table 2:** Attack performance of different adversarial camouflages in the digital domain against different detectors. Evaluated by MR (%) and P@0.5 (%).

Method	Object Detector													
	YOLOv5		YOLOv8		YOLO11		Faster R-CNN		Mask R-CNN		SSD		RT-DETR	
	MR ↑	P@0.5 ↓	MR ↑	P@0.5 ↓	MR ↑	P@0.5 ↓	MR ↑	P@0.5 ↓	MR ↑	P@0.5 ↓	MR ↑	P@0.5 ↓	MR ↑	P@0.5 ↓
Raw	28.78	78.39	29.2	77.14	19.75	77.18	33.30	74.60	30.40	63.70	37.40	68.90	13.09	83.43
Random noise	41.87	69.88	49.20	70.32	38.67	69.77	49.00	46.30	46.40	41.10	45.90	52.20	34.66	73.05
CAMOU	46.63	65.92	47.97	69.70	40.77	65.68	50.70	47.30	44.70	44.40	50.40	43.90	40.44	70.35
ER	40.99	75.23	42.82	75.22	32.07	72.37	46.00	53.50	38.50	55.60	44.70	53.70	31.90	74.85
DAS	41.93	74.35	42.90	70.19	28.10	72.62	43.00	56.20	42.20	50.60	43.90	56.10	31.43	72.15
FCA	79.63	28.44	76.18	33.34	73.83	30.16	79.40	7.30	75.90	5.30	77.60	7.10	77.57	38.48
MFA	77.13	31.05	71.97	37.26	68.23	38.00	82.00	5.30	76.70	9.10	78.10	6.50	82.63	28.54
DTA	39.47	74.52	42.41	74.80	32.73	72.57	45.70	54.30	38.00	56.30	44.30	54.90	31.79	73.31
ACTIVE	46.83	66.99	47.16	71.17	37.89	70.49	52.50	44.90	43.10	47.80	48.70	54.90	42.07	68.49
MCC	53.60	64.16	58.30	61.34	42.57	64.71	46.10	44.00	53.70	35.20	56.50	32.00	39.63	66.53
RAUCA	85.80	18.25	78.23	27.44	70.03	31.21	82.40	4.10	75.60	5.70	87.00	2.10	92.07	11.56
TACT-person	70.67	40.28	66.87	44.86	58.17	47.02	81.20	5.70	72.50	10.50	88.30	3.30	81.87	30.09
TACT-bird	73.93	36.18	77.10	38.02	59.00	49.15	82.80	4.70	74.50	10.00	91.80	1.0	78.47	32.96
TACT-traffic	69.30	45.77	65.87	47.81	57.53	54.99	75.40	11.30	61.90	15.80	74.20	9.0	84.80	26.57

**Table 3:** Targeted attack effect of adversarial camouflages in the digital domain. Evaluated by TASR (%).

Method	Object Detector							
	YOLOv5	YOLOv8	YOLO11	Faster R-CNN	Mask R-CNN	SSD	RT-DETR	Mean TASR
MCC	36.47	44.82	21.70	31.11	32.26	68.83	29.19	37.77
TACT-person	**50.02**	44.60	35.16	**67.13**	**70.22**	**83.86**	12.37	**51.91**
TACT-bird	45.54	**66.29**	**39.86**	32.83	12.07	69.02	9.17	32.95
TACT-traffic	34.17	41.41	31.69	58.79	56.42	65.31	**62.88**	50.10

Notes: Bold values denote the optimal performance, underlined values denote the suboptimal performance.

**Table 4:** Targeted attack effect of adversarial camouflages in the digital domain. Evaluated by TCER (%).

Method	Object Detector							
	YOLOv5	YOLOv8	YOLO11	Faster R-CNN	Mask R-CNN	SSD	RT-DETR	Mean TCER
MCC	51.33	53.63	32.17	39.10	33.80	76.87	29.40	45.19
TACT-person	62.83	55.97	**48.67**	**68.53**	**71.63**	**88.77**	14.03	**58.63**
TACT-bird	**63.20**	**70.93**	48.40	35.77	16.73	77.37	12.13	46.36
TACT-traffic	52.93	52.37	45.77	60.33	57.70	74.80	**63.37**	58.18

Notes: Bold values denote the optimal performance, underlined values denote the suboptimal performance.

**Table 5:** Attack performance of different adversarial camouflages in the physical domain against detectors. Evaluated by mAP50-95 (%).

Method	Object Detector											
	YOLOv5						YOLOv8					
	0°	30°	45°	60°	90°	All	0°	30°	45°	60°	90°	All
Raw	98.93	98.82	98.22	91.39	54.06	83.11	98.73	98.84	97.54	45.23	53.01	81.53
FCA	94.79	93.13	81.39	43.48	**0.00**	49.92	97.98	95.93	65.50	9.01	3.15	50.08
RAUCA	94.20	**17.77**	**24.75**	20.38	**0.00**	31.66	93.56	**8.70**	**2.68**	**0.00**	**0.00**	28.84
TACT-person	88.47	59.33	51.11	13.94	4.51	29.90	97.64	67.14	22.13	**0.00**	**0.00**	37.38
TACT-bird	**88.34**	52.94	24.92	**11.52**	**0.00**	**26.18**	**87.83**	1.77	2.79	**0.00**	**0.00**	**21.66**

Notes: Bold values denote the optimal performance, underlined values denote the suboptimal performance.

**Table 6:** Attack performance of TACT variants under different detectors and angles (evaluated by TASR (%) and TCER (%)).

Method	Mask R-CNN											
	0°		30°		45°		60°		90°		All	
	TASR	TCER	TASR	TCER	TASR	TCER	TASR	TCER	TASR	TCER	TASR	TCER
TACT-person	3.42	4.24	7.21	11.97	76.15	76.79	24.49	66.96	88.60	88.70	41.40	49.12
TACT-bird	0	14.66	2.61	3.49	17.24	36.28	9.09	27.27	19.66	19.66	9.70	20.14
	**YOLOv5**											
	**0°**		**30°**		**45°**		**60°**		**90°**		**All**	
	**TASR**	**TCER**	**TASR**	**TCER**	**TASR**	**TCER**	**TASR**	**TCER**	**TASR**	**TCER**	**TASR**	**TCER**
TACT-person	0	9.32	0	52.14	23.53	76.78	0	97.32	61.11	75.65	19.12	61.67
TACT-bird	2.15	21.55	8.77	54.78	3.17	46.02	0	64.55	10.53	53.85	4.81	47.99

**Table 7:** The impact of supervision redirection on adversarial camouflage performance.

Detector	Raw_R	Raw_mAP50-90	TACT-Car_R	TACT-Car_mAP50-90
YOLOv5	71.22	59.52	**80.07**	**62.59**
YOLOv8	70.80	57.55	**71.57**	**60.76**
YOLO11	80.25	63.21	**81.67**	**68.41**
Faster R-CNN	66.70	**49.00**	**68.30**	46.00
Mask R-CNN	69.60	**45.80**	**73.70**	44.10
SSD	62.60	**47.10**	**69.60**	45.50
RT-DETR	**86.91**	**70.71**	73.43	58.50

## Data Availability

Data related to the current study are available from the corresponding author upon request.
